# In Vitro Interactions Between Bacteriophages and Antibacterial Agents of Various Classes Against Multidrug-Resistant Metallo-β-Lactamase-Producing *Pseudomonas aeruginosa* Clinical Isolates

**DOI:** 10.3390/ph18030343

**Published:** 2025-02-27

**Authors:** Paschalis Paranos, Sophia Vourli, Spyros Pournaras, Joseph Meletiadis

**Affiliations:** 1 Clinical Microbiology Laboratory, Attikon University Hospital, Medical School, National and Kapodistrian University of Athens, Rimini 1, Haidari, 12462 Athens, Greece; pasxalisparanos@hotmail.gr (P.P.); svourli@med.uoa.gr (S.V.); spournaras@med.uoa.gr (S.P.); 2 Institute of Biosciences and Applications, National Center for Scientific Research “Demokritos”, Patr. Gregoriou E & 27 Neapoleos Str, 15341 Agia Paraskevi, Greece

**Keywords:** bacteriophages, antibacterial agents, metallo-β-lactamases, checkerboard

## Abstract

**Background:** Combination therapy with antibiotics and phages has been suggested to increase the antibacterial activity of both antibiotics and phages. We tested the in vitro activity of five antibiotics belonging to different classes in combination with lytic bacteriophages against multidrug-resistant metallo-β-lactamase (MBL)-producing *Pseudomonas aeruginosa* isolates. **Material/Methods:** A total of 10 non-repetitive well-characterized MBL-producing *P. aeruginosa* isolates (5 NDM, 5 VIM) co-resistant to aminoglycosides and quinolones were used. Phage–antibiotic interactions were assessed using an ISO-20776-based broth microdilution checkerboard assay in 96-well microtitration plates. Two-fold dilutions of colistin (8–0.125 mg/L), ciprofloxacin, meropenem, aztreonam, and amikacin (256–4 mg/L) were combined with ten-fold dilutions of five different phages (5 × 10^9^–5 × 10^0^ PFU/mL) belonging to *Pakpunavirus*, *Phikzvirus, Pbunavirus*, and *Phikmvvirus* genus. Plates were incubated at 35 ± 2 °C for 24 h, and the minimum inhibitory concentration of antibiotics (MIC_A_) and phages (MIC_P_) were determined as the lowest drug and phage concentration, resulting in <10% growth based on photometric reading at 550 nm. Interactions were assessed based on the fractional inhibitory concentration index (FICi) of three independent replicates and clinical relevance based on the reversal of phenotypic resistance. The statistical significance of each drug alone and in combination with phages was assessed using GraphPad Prism 8.0. **Results:** Synergistic and additive interactions were found for 60–80% of isolates for all drugs. FICis were statistically significantly lower than 0.5 for colistin (*p* = 0.005), ciprofloxacin (*p* = 0.02), meropenem (*p* = 0.003), and amikacin (*p* = 0.002). Interactions were found at clinically achievable concentrations for colistin, meropenem, and amikacin, and a reversal of phenotypic resistance was observed for most strains (63–64%) for amikacin and meropenem. Antagonism was found for few isolates with all antibiotics tested. Phage vB_PaerM_AttikonH10 and vB_PaerP_AttikonH4 belonging to *Phikzvirus* and *Phikmvvirus* genus, respectively, showed either synergistic (FICi ≤ 0.35) or additive effects with most antibiotics tested. **Conclusions:** Synergy was observed for most drugs and phages with amikacin, showing strong synergy and reversal of phenotypic resistance against most isolates. Taking into account the wide utility of jumbo phages obtained, the findings of vB_PaerM_AttikonH10 in combination with different classes of antibiotics can enhance the activity of currently ineffective antibiotics against MBL-producing *P. aeruginosa* isolates.

## 1. Introduction

*Pseudomonas aeruginosa* is an opportunistic bacterium that can cause wide range of complex recurrent infections, with high morbidity and mortality, particularly in immunocompromised individuals [1]. According to the European Center for Disease Prevention and Control (ECDC), in Greece, 45.5% of the isolates had combined resistance to at least three classes of antibiotics, making it the highest rate among other European countries (https://atlas.ecdc.europa.eu/public/index.aspx). It has recently been found that 82.3% of all carbapenem-resistant infections were caused by *P. aeruginosa* or *Acinetobacter baumannii*; whereas, only 17.7% were caused by *Klebsiella pneumoniae* or *Escherichia coli* [2]. Moreover, metallo-β-lactamase (MBL)-producing *P. aeruginosa* isolates pose a serious therapeutic challenge, as newer β-lactam-β-lactamase inhibitors (including ceftolozane/tazobactam, ceftazidime/avibactam ± aztreonam, meropenem/vaborbactam, and imipenem/relebactam) are inactive against these pathogens [3]. For that reason, taking decisive actions is of primary importance for combating against infections caused by MBL-producing *P. aeruginosa* isolates.

As the development of new antibiotics has reached its limits, and the emergence of resistance to antibiotics is inevitable, alternative therapeutic approaches are needed for treating infections caused by multidrug-resistant (MDR) and, in particular, MBL-producing *P. aeruginosa* isolates. Phage therapy may fulfill this need, as lytic phages can distribute to different body compartments, rapidly kill host bacteria upon contact, and multiply at the site of infection [4]. Despite the fact that phages have been used as an anti-infective agent for more than a century [5], interest in using them for treating MDR infections has resurfaced with the rise of antibiotic resistance [6]. However, one of the major limitations of phage therapy is the development of resistance, particularly against monophage therapy, albeit with some fitness costs, like reduced virulence and resensitization to antibiotics [7]. Resistance mechanisms to antibiotics may differ from resistance mechanisms to phages. Thus, employing phages as combination therapy with antibiotics, rather than on their own, may be a more pertinent approach with wider application [8].

In vitro studies have suggested the effectiveness of phages in combination with different classes of antibiotics [9,10,11]. The combination of either front-line or broad-spectrum antibiotics was found to act synergistically with *Pseudomonas* phage on planktonic cells with more than 4-log kill found between sublethal concentrations of cefotaxime, meropenem, tetracycline, and gentamicin, when combined with phages at a concentration of 10^3^ PFU/mL against a resistant *P. aeruginosa* isolate [11]. However, the combination of ciprofloxacin and meropenem with phages using bacteria in a planktonic phase showed antagonistic or indifferent interactions at 4 h and 22 h, respectively [12]; whereas, in another study, synergistic effects were observed [11]. However, there are no data concerning MBL-producing *P. aeruginosa* isolates, which are pathogens that are of great concern.

We therefore studied the interaction between antibiotics from five distinct classes, with different mechanisms of actions (colistin, meropenem, ciprofloxacin, amikacin, and aztreonam), and phages belonging to different genera against MDR MBL-producing *P. aeruginosa* isolates using a broth microdilution method.

## 2. Results

### 2.1. In Vitro Activity of Drugs and Phages Alone

The median (range) MIC_A_s and MIC_P_s against MDR MBL-producing *P. aeruginosa* strains are shown in Table 1. Considering EUCAST clinical breakpoints, 1/10, 9/10, 8/10, and 5/10 were resistant to colistin, amikacin, meropenem, and aztreonam, respectively.

### 2.2. In Vitro Activity of Antibiotic–Phage Combination

The number of strains and phages with synergistic, additive, and antagonistic interactions together with the FICi, MIC_A_ and MIC_P_ alone and in combination, are shown in Table 2 for each drug. Two previously isolated phages vB_PaerP_AttikonH4 and vB_PaerM_AttikonH10, which belong to *Phikmvvirus* and *Phikzvirus* genus, respectively, showed either synergistic or additive interactions with all antibiotics tested. In particular, vB_PaerM_AttikonH10 showed significant synergistic effects (FICi ≤ 0.13) with all antibiotics tested except colistin and aztreonam, where additive effects were found. MIC_A_ in synergistic combinations were substantially reduced up to six two-fold dilutions within the clinically achievable concentrations. For meropenem and amikacin, the median MIC_A_ was >256 and 128 mg/L and dropped down to 8 mg/L after the addition of phage;. Even for antagonistic combinations MIC_A_ was reduced as antagonism was due to an increase in MIC_P_. The % of strains with synergistic, additive, and antagonistic interactions are shown in Table 3. The differences among the three independent replicates were <10%.

Synergy was found for 40–60% of isolates for amikacin, ciprofloxacin, meropenem, and colistin; whereas, for aztreonam, additivity was found for most (70%) isolates. Synergistic interactions were strong with FICis ranging from 0.04 to 0.35 for all isolates. For the isolates with synergistic interactions, the FIC_min_s for colistin, ciprofloxacin, meropenem, and amikacin were statistically significantly lower than 0.5 for all isolates (*p* ≤ 0.02). The MIC_A_ and MIC_P_ in synergistic combinations were significantly lower than the MIC_A_ and MIC_P_ alone for all drugs and isolates (*p* ≤ 0.03). Antagonism was found for 20–40% of strains with all drugs, and it was strong for ciprofloxacin, as the median (range) FICi was 1000 (11–1001). For the isolates with antagonistic interactions, the FICis for ciprofloxacin only was significantly higher than 4 (*p* = 0.02). The MIC_A_ in antagonistic combinations was not significantly higher than the MIC_A_ alone for all drugs (*p* > 0.05), where the MIC_P_ increase was significant for ciprofloxacin, amikacin, and aztreonam (*p* < 0.05).

### 2.3. Clinical Relevance of Interactions

**Figure 1:**
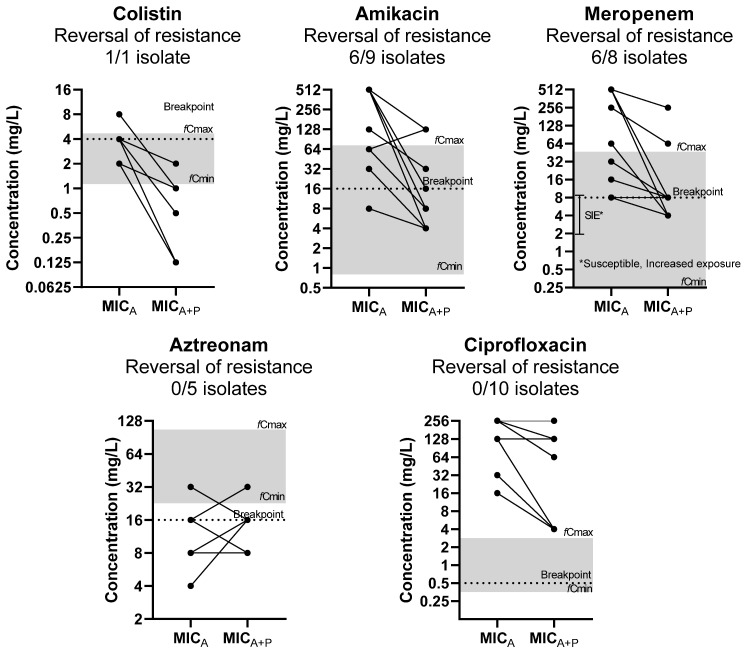
Effect of phages on MIC of antibiotics (MIC_A_) in relation to clinically achievable serum concentration and clinical breakpoints (reversal of phenotypic resistance). The median MIC_A_ of three replicates of each isolate is presented on the left and the median MIC_A_ of three replicates in combination with the phage (MIC_A+P_) on the right. Each line may represent more than 1 isolate. Gray zone represents the area of clinically achievable free drug concentrations in human blood. Dotted line represents the EUCAST breakpoint of each drug.

The reversal of phenotypic resistance was found for interactions to amikacin in 6/9 resistant isolates, to meropenem in 6/8 resistant isolates, and to colistin in 1/1 resistant isolates based on EUCAST clinical breakpoints (Figure 1) [13]. Regarding meropenem, 6/8 were susceptible to increased exposure, according to EUCAST guidelines. The reversal of phenotypic resistance was statistically significant (*p* < 0.05), as the MICs of drugs was significantly higher than corresponding clinical breakpoints when tested alone but not when tested in combination. No reversal of phenotypic resistance was found for aztreonam and ciprofloxacin, despite the MIC reduction.

The synergistic/additive interactions were found at clinically achievable free drug concentrations for all drugs, except for ciprofloxacin. In particular, for antibiotics, the concentrations of synergistic interactions with phages were observed at clinically achievable levels for amikacin, meropenem, and colistin in 80%, 80%, and 83% of strains, respectively. For phages, synergistic/additive combinations with antibiotics were observed at clinically achievable phage concentrations for amikacin, ciprofloxacin, meropenem, colistin, and aztreonam in 38%, 17%, 25%, 0%, and 43% of strains, respectively.

The antagonistic interactions were due to the increase in MIC_P_ when phages were combined with antibiotics, as the MIC_A_ was the same or even reduced in combinations with phages. These interactions were observed at clinically achievable antibiotic concentrations for amikacin, aztreonam and colistin in all strains and for meropenem in most strains whereas for ciprofloxacin antagonistic interactions were observed at clinical non-achievable concentrations. For phages, their concentrations at antagonistic combinations with antibiotics were not observed at clinically achievable concentrations for all drugs.

## 3. Discussion

Synergistic interactions were found between the phages and antibiotics of different classes with most synergistic combinations found for 40–60% strains with amikacin, ciprofloxacin, meropenem, and colistin. Synergistic interactions were strong, as the FICi for all isolates tested were 0.04–0.35). For all drug–phage combinations, except ciprofloxacin, interactions were found at clinically achievable concentrations for both phages and antibiotics; whereas, a reversal of phenotypic resistance for 64% and 63% of resistant isolates for amikacin and meropenem was found, respectively. In colistin, a reversal of phenotypic resistance was observed in 1/1 isolate that was resistant, while an MIC_A_ reduction was observed in most of the strains. Antagonism was also found for few strains (20–40%), usually with high MIC_A_ to antibiotics, and it was due to a ≥100-fold increase in the MIC_P_ in the presence of antibiotics. Interestingly, vB_PaerM_AttikonH4 and vB_PaerM_AttikonH10, which belong to *Phikmvvirus* and *Phikzvirus* genus, respectively, remarkably showed either synergistic or additive but not antagonistic interactions with all antibiotics tested. Phages belonging to the jumbo *Phikzvirus* genus are particularly useful in phage therapy [14,15,16,17] because of their broad spectrum of lytic activity and resistance to treatment with different agents; whereas, phages belonging to the *Phikmvvirus* genus showed remarkable effectiveness in combination with antibiotics against *P. aeruginosa* [18].

Several mechanisms for the synergy between antibiotics and phages have been proposed. These include the elongation and filamentation of cells by antibiotics, the increase in plaque size, the acceleration of phage amplification, and the enhancement of burst size; the reduction in phage and/or antibiotic-resistant mutant appearance; the increased susceptibility to antibiotics due to the presence of the phage; and the depolymerization of bacterial polysaccharides by phage enzymes (glycan depolymerases), which increase antibiotic diffusion and cell penetration [8]. However, as phages are using the bacterial machinery to produce virions, it was previously found that antibiotics targeting bacterial protein synthesis (e.g., aminoglycosides) reduced the lytic activity of bacteriophage against *A. baumannii*, *Staphylococcus aureus* and *Salmonella typhimurium* [19]. Our results were in concordance with previous findings from other in vitro and in vivo experiments using a combination therapy of phages and antibiotics against *P. aeruginosa* isolates. Concerning *P. aeruginosa*, it has been found that amikacin–phage dynamic antagonism has not been noticed at the end point of 22 h [12]. Ιn the present study, amikacin demonstrated 60% synergy, especially for isolates with MIC_A_ ≤ 64 mg/L, while for isolates with MIC_A_, > 64 mg/L had an either additive or antagonistic impact. Ciprofloxacin has been the most utilized antibacterial agent combined with phages against few (2–6 isolates) *P. aeruginosa* isolates and synergy was found [20,21,22]. Ciprofloxacin demonstrated synergistic effects for 50% of the isolates in the present study. As bacterial DNA gyrase, which is the mechanism of action of ciprofloxacin, is used by the phage [23], one would expect antagonism between antibiotics inhibiting DNA gyrase and phages. Here, synergistic interactions were observed in isolates with MIC_A_ < 128 mg/L, while antagonistic interactions were noticed for isolates with MIC_A_ ≥ 128 mg/L. In another study, the phage–ciprofloxacin combination against MDR isolates reduced MICs five two-fold dilutions (2 to 0.06 mg/L) with a subsequent reversal of phenotypic resistance, while less promising results have been found for the combination with meropenem that reduced MICs by one two-fold dilution (32 to 16 mg/L) [24]. Contrary to the previous study, we did not observe a reversal of phenotypic resistance to ciprofloxacin, despite the synergistic interactions. This discrepancy may be due to the very high MICs of the isolates in the present study compared to the previous study (median MIC_A_ 256 vs. 2 mg/L, respectively). Concerning colistin, it was shown to interfere with bacteriolytic and virion production activities, even in the presence of 1× MIC_A_. Phage use LPS as their surface receptor, while colistin damages cell membrane by disrupting cation bridges between LPS molecules. This could explain the low synergistic effects of that antibiotic on bacteriophage infection activity [1]. The efficacy of aztreonam in combination with phages has not been demonstrated against planktonic cells, but it has been found in an in vitro static biofilm model that combination therapy resulted in a significant reduction in biofilm mass compared to phage treatment or antibiotics alone against the *P. aeruginosa* PA01 reference strain [25]. In the present study, aztreonam conferred additivity against the isolates tested.

The checkerboard broth microdilution method is the standard approach for evaluating drug combinations by estimating the reduction in MIC_A_ in combination relative to the MIC_A_s of the antimicrobial agents alone [26]. When both antibacterial agents’ MIC_A_s are lowered by more than two two-fold dilutions, synergy is concluded. Considering that, we used the same approach to evaluate phage–antibiotic interactions. However, MIC_A_ reduction may be clinically irrelevant if it happens at supraphysiological concentrations, or if MIC_A_s are not reduced below the susceptibility threshold. Amikacin and meropenem showed comparable FIC indices and showed synergy at clinically achievable free drug concentrations. However, amikacin was found to reverse phenotypic resistance in more strains, making it the best candidate for phage–antibiotic combination against MBL-producing *P. aeruginosa* isolates. Aztreonam concentrations in additive combinations were also observed at clinically achievable levels, however, without reversing phenotypic resistance in all isolates. Such an analysis cannot be performed on phage concentrations, since there are no essential threshold concentrations or breakpoints associated with clinical outcome.

Surprisingly, antagonistic interactions were also observed for some strains at drug concentrations at 1x to 0.125xMIC_A_, mainly due to an increase in the MIC_P_ of phages, indicating that antibiotics may interact antagonistically at high phage concentrations. Recent studies show antagonism among phages in a cocktail when used at high titers against *K. pneumoniae* [4]. Moreover, it has been found that some phage cocktails demonstrated a narrower host range than the sum of each individual phage alone [27]. This could be due to competition between phages, so that the coinfection of the same host bacterial cell may result in increased competition for limited cellular resources, lowering the fitness of an individual phage [27]. Phage–receptor site sharing or abortive infection pathways may potentially result in antagonistic interactions [28]. It was suggested that, for phage–antibiotic combination therapy, antagonistic interactions can be affected by (i) phage parameters and antibiotic class, (ii) the concentration and ratios of phage and the antibiotic used in combination, (iii) the simultaneous or sequential order of application, (iv) treatment lengths, and (v) host environment [29].

Among the limitations of the present study are the small number of isolates and phages tested. Bactericidal interactions and the emergence of resistance beyond 24 h was not explored. Interactions were assessed in a static model; whereas, in vivo drug and phage concentrations change over time. Finally, the impact of serum on in vitro interactions was not studied.

Despite the extensive research that has been performed over in recent years on the combination of phages with antibiotics, the data mainly concern either in vitro biofilm or in vivo models for a few drugs, phages, and isolates. In particular, there are no data on in vitro interaction between different phages and antibiotic classes against the planktonic phase of carbapenemase-producing *P. aeruginosa* isolates. Thus, the present study provides novel insights on phage therapy against MBL-producing *P. aeruginosa* isolates, particularly those with high level of resistance to various classes of antibiotics. From the methodological point of view, the results were analyzed using the standard checkerboard broth microdilution method introducing the concept of the MIC of phages, and the data were analyzed with the FIC index, assessing both synergistic and antagonistic interactions with statistical terms. Although synergistic interactions were obtained using phages in combination with various classes of antibiotics, amikacin or meropenem seems to be the most promising candidates for combination therapy, as drug concentrations in the combination were within clinically achievable blood concentrations, and there was a reversal of the phenotypic resistance for most strains. Our results support the concept of combination therapy with antibiotics with phages against MBL-producing *P. aeruginosa* isolates, where therapeutic options are not available. In future studies, we aim to employ the most synergistic combinations of phages and antibiotics in an in vitro dynamic model and in in vivo animal models, in order to verify current findings.

## 4. Material and Methods

### 4.1. Bacterial Strains

A total of 10 non-repetitive well-characterized MBL-producing *P. aeruginosa* isolates (5 NDM and 5 VIM) co-resistant to carbapenems and fluoroquinolones (100%) and aminoglycosides (90%) were used [30]. Non-clonality was verified by Whole-Genome Sequencing [31], and Multilocus Sequency Typing (MLST) analysis was performed on all isolates by web-based methods based on Whole Genome Sequencing data [32]. Bacterial strains were stored at −80 °C in Trypticase Soy Broth (TSB) (Oxoid, UK) supplemented with 20% glycerol until further use. In order to recover the strains, a small amount was cultured in MacConkey agar No. 3 (MAC) (Oxoid, UK) plates and incubated at 37 °C for 24 h. Presumptive MBLs were previously confirmed via the Combined Disk Synergy Test (CDST) [33], using 0.1 M EDTA, and multiplex PCR was used in order to screen the expression of MBL genes [34].

### 4.2. Bacteriophages

Five distinct phages (vB_PaerM_AttikonH2, vB_PaerP_AttikonH4, vB_PaerM_AttikonH5, vB_PaerM_AttikonH7, and vB_PaerM_AttikonH10) specific against MBL-producing *P. aeruginosa* isolates, belonging to the *Pakpunavirus* (*n* = 2), *Phikzvirus* (*n* = 1), *Pbunavirus* (*n* = 1), and *Phikmvvirus* (*n* = 1) genus, were previously isolated from wastewater samples from the Psyttaleia region [35]. Phages have been selected from a larger collection based on heterogeneity and the ability to lyse MBL-producing *P. aeruginosa* isolates. Isolated phages were purified by triplicate single plaque transfer and propagated until homologous plaques were obtained [36]. Their titer was determined by single-layer agar [37], and their stocks were kept in Luria Bertani broth (LB) (AppliChem, according to Miller, Athens, Greece) at 4 °C until further use.

### 4.3. Antibacterial Drugs and Media

Five antibiotics were selected based on the mechanism of action and their wide application against infections caused by *P. aeruginosa*, including two beta-lactams the carbapenem meropenem and monobactam aztreonam targeting the cell wall, colistin targeting the cell membrane, the aminoglycoside amikacin targeting protein synthesis, and the fluoroquinolone ciprofloxacin targeting protein synthesis. Pure powders were purchased from Sigma Aldrich chemical company (St. Louis, MO, USA), except for colistin, which was kindly provided by Pfizer Inc. (Philadelphia, PA, USA). Cation-adjusted Mueller–Hinton broth II (caMHB) (Difco, Athens, Greece) supplemented with 25 mg/L calcium and 12.5 mg/L magnesium was used for antimicrobial susceptibility testing and checkerboard assays.

### 4.4. In Vitro Susceptibility of Drugs and Phages Alone

The minimal inhibitory concentration of antibiotics (MIC_A_) were determined by broth microdilution, according to the ISO-20776 method [38]. The working stock solutions of antimicrobials were freshly prepared in caMHB. Isolates were tested against colistin (8–0.125 mg/L), amikacin (256–4 mg/L), meropenem (256–4 mg/L), aztreonam (256–4 mg/L), and ciprofloxacin (256–4 mg/L) in two-fold dilutions. Each tray contained wells with 50 μL of serial two-fold dilutions of two times the final concentration of antimicrobial drugs and a negative and a positive growth control in every run. Once the microdilution trays were made, they were stored at −70 °C up to 1 month until use. On the day of the experiment, trays were thawed, inoculated with 50 μL 1 × 10^6^ CFU/mL bacterial suspension prepared from a 24 h culture grown on a MAC, and incubated at 35 ± 2 °C in ambient air, in order to have final concentration of 5 × 10^5^ CFU/mL. Each set of MIC_A_ determinations included two ATCC control strains: *E. coli* (ATCC 25922) and *P. aeruginosa* (ATCC 27853). After 18–24 h of incubation, MIC_A_s were assessed photometrically at OD550 (Biorad, PR 4100) after agitation (220 rpm) as the lowest drug concentration causing >90% inhibition of bacterial growth. Three independent replicates were tested between different days in order to check the interday variability.

The minimum inhibitory concentrations of phages (MIC_P_) were determined by the broth microdilution method. In short, phages used in the current study were ten-fold serially diluted in caMHB, and 50 μL was placed in 96-well plates (ThermoFischer, Cat. No. 167008) in order to obtain final concentrations of 5 × 10^9^–5 × 10^0^ PFU/mL (MOI ranging 10^3^–10^−7^) after bacterial inoculation. Because the lowest concentration in the microplate is closed to 1 PFU/well, we have quantitated phage concentrations using single-layer agar spot assay [37] in several wells with 5 × 10^0^ PFU/mL, and we found concentrations 1–7 PFU/well for different phages and replicates. Bacterial inoculum was prepared from a 24 h culture grown on a MAC adjusted to 0.5 McFarland standard, which was diluted 1:100 in caMHB, and 50 μL was added into each well of already inoculated phages, ending up in a final bacterial concentration of 5 × 10^5^ CFU/mL. A negative and a growth control were used for each isolate and phage combination. Plates were incubated at 35 ± 2 °C for 24 h, and MIC_P_s were assessed photometrically at OD_550_ after agitation (220 rpm) for 1 min as the lowest phage concentration causing >90% inhibition of bacterial growth. Three independent replicates were performed.

### 4.5. Combination of Antibacterial Drugs with Phages

**Figure 2:**
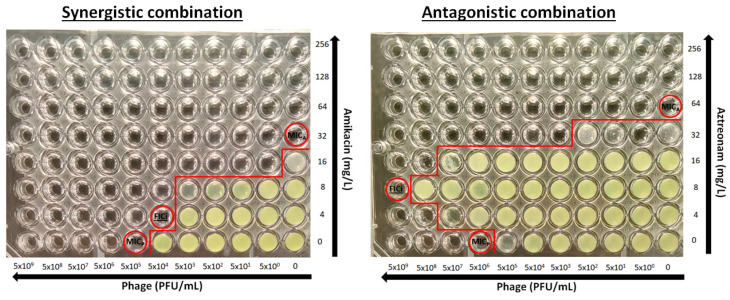
Examples of a synergistic and antagonistic combination. MIC_A_ and MIC_P_ are the minimal inhibitory concentrations of antibiotics and phages, respectively and FICi is the fractional inhibitory concentration index (circled wells). Red line separates wells with and without growth.

The determination of the activity of the phage–antibiotic combination was performed using the checkerboard assay (Figure 2). Briefly, antibacterial drugs were two-fold serially diluted in caMHB, and 25 μL was inoculated to each column (A–G) in a 96-well plate (ThermoFischer, Cat. No. 167008) at concentrations 4-times higher than the final concentrations after phage addition and bacterial inoculation and placed at −70 °C. Phages were serially diluted 1:10 in caMHB in order to obtain 4× the final phage concentrations of 5 × 10^9^–5 × 10^0^ PFU/mL. On the day of the experiment, plates were thawed properly at room temperature, and 25 μL of phage suspension was added in each row of the 96-well microplate. Each plate consisted of two-fold decreasing concentrations of the drug in rows A to G, with row H containing no drug and ten-fold decreasing concentrations of phages in columns 1 to 10, with column 11 and 12 containing no phage (Figure 1). Wells H11 and H12 were used as a growth control. A bacterial suspension of 0.5 McFarland concentration was prepared from a 24 h culture, and 50 µL of a 1:100 dilution in caMHB was added to the wells of the plate, which were already inoculated with phage, resulting in a final concentration of 5 × 10^5^ CFU/mL. The plates were incubated at 35 ± 2 °C for 24 h and the MIC_A_s and MIC_P_s alone and in combination were determined photometrically at OD_550_ after agitation (220 rpm) as the lowest concentration with >90% inhibition of bacterial growth. Single wells with bacterial growth that did not follow a concentration-dependent decrease in growth were excluded from the analysis. All combinations were tested in triplicate.

### 4.6. Fractional Inhibitory Concentration Index (FICi)

The FICi was calculated using the following equation for each antibiotic (A)-phage (P) combination:

$$FICi={FIC}_{A}+{FIC}_{P}=\frac{{MIC}_{A+P}}{{MIC}_{A}\;alone}+\frac{{MIC}_{P+A}}{{MIC}_{P}\;alone}$$

where MIC_A_ alone and MIC_P_ alone are the MIC of drug alone and the MIC of phage alone, respectively; and MIC_A+P_ and MIC_P+A_ are the MIC of drug in presence of phage and the MIC of phages in the presence of the drug at iso-effective combinations (>90% inhibition), respectively. To capture synergistic and antagonistic interactions, the FIC_min_ and FIC_max_ were calculated for each strain and each replicate for all antibiotics tested. Drug and phage concentrations in synergistic, additive, and antagonistic combinations were determined.

### 4.7. Clinical Relevance

Since in vitro growth inhibitory drug concentrations were determined in media without serum, and in vivo drugs are bound in serum proteins like albumin, in vitro concentrations should be compared with in vivo free drug concentrations taking into account the protein binding rate if each drug [39]. Thus, in order to assess the clinical relevance of in vitro interactions, the percentage of strains where the interactions were at or lower than clinically achievable free drug concentrations in human blood after the administration of established doses of the drugs were calculated, i.e., *f*C_max_–*f*C_min_ range 2.2–0.91 mg/L for colistin [40], 66.99–0.93 mg/L for amikacin [41], 49–0.2 mg/L for meropenem [42], 106.48–17.99 mg/L for aztreonam [43], and 2.88–0.31 mg/L for ciprofloxacin [44]. For phages, blood levels ranged from 4 × 10^3^–1.8 × 10^4^ PFU/mL after the intravenous administration of 4 × 10^9^ PFU q8h [45].

Finally, the percentage of strains whose phenotypic resistance was reversed was defined as the number of resistant strains that became susceptible in the presence of the phages, i.e., MIC_A+P_ ≤ 4 mg/L, ≤16 mg/L, and ≤2 mg/L for colistin, amikacin, and meropenem and susceptible increased exposure, i.e., ≤8, ≤0.5, and ≤16 mg/L for meropenem, ciprofloxacin, and aztreonam, respectively [13]. The reversal of phenotypic resistance was calculated only for resistant isolates based on the MIC_A_ and the MIC_A+P_ for both synergistic and additive interactions. The susceptible isolates (1 for amikacin, 9 for colistin, 2 susceptible increased exposure for meropenem and 5 for aztreonam) were excluded from the analysis.

### 4.8. Statistical Analysis

The results for each drug in combination with phages were analyzed by assessing the statistical significance. In order to assess whether synergy and antagonism was statistically significant for each strain individually and in total for all strains, the differences among replicates (technical variation) and strains (biological variation) of FICis from 0.5 for strains with synergy and FICis from 4 for strains with antagonism were assessed with one sample *t*-test after the log_2_ transformation of the FICi values [46]. In any other case, additivity was claimed. Drug and phage concentrations at the synergistic, additive, and antagonistic combinations were determined. Furthermore, in order to assess whether the decrease and increase in drug and phage concentrations in synergistic and antagonistic combinations, respectively, were statistically significant, drug and phage concentrations alone were compared with those in combination using a paired *t*-test for each antibiotic after the log_2_ transformation of drug concentrations and log_10_ transformation of phage concentrations. Finally, the significance of the reversal of phenotypic resistance was assessed by comparing drug concentrations in synergistic and additive combinations with the corresponding clinical breakpoints of each drug using one sample t-test after the log_2_ transformation of drug concentrations. A *p* value <0.05 was considered significant. GraphPad Prism 8.0.2 was used for the statistical analysis of the results.

## 5. Conclusions

Synergistic interactions were found between the phages and antibiotics of different classes with most synergistic combinations found with amikacin, ciprofloxacin, meropenem, and colistin. For all drug–phage combinations, except ciprofloxacin, interactions were found at clinically achievable concentrations for both phages and antibiotics; whereas; the reversal of phenotypic resistance was found for most resistant strains with amikacin and meropenem. Antagonism was also found for a few strains at higher phage concentrations and antibiotic concentrations lower than the MIC. The combination of jumbo phage vB_PaerM_AttikonH10 with different antibiotics demonstrated high synergistic effects (FICi ≤ 0.13) and was able to reduce up to six two-fold dilutions (from >256 down to 8 mg/L) with the MIC_A_ of isolate within the clinically achievable drug concentrations. This observation makes vB_PaerM_AttikonH10 a good candidate that can efficiently enhance the action of several antibiotics against MBL-producing *P. aeruginosa* clinical isolates.

## Figures and Tables

**Table 1:** Median (range among replicates) minimum inhibitory concentrations of phages (MIC_P_) and minimum inhibitory concentrations of antibiotics (MIC_A_) colistin, meropenem, ciprofloxacin, amikacin, and aztreonam against 10 MDR MBL-producing *Pseudomonas aeruginosa* clinical isolates. For each isolate, the resistance mechanism, multilocus sequence type (MLST), as well as the phage used and its taxonomy, are presented.

Isolates	Resistance Mechanism ^a^	MLST	Phages	Taxonomy	MIC_P_ (PFU/mL)	MIC_A_ (mg/L)				
						Colistin	Meropenem	Ciprofloxacin	Amikacin	Aztreonam
AUHB215	NDM	ST773	vB_PaerM_AttikonH5	*Pakpunavirus*	5 × 10^6^ (5 × 10^5^–5 × 10^7^)	4 (1–4)	>256 (>256–>256)	256 (128–256)	>256 (>256–>256)	8 (8–8)
AUHB217	NDM	ST308	vB_PaerM_AttikonH7	*Pbunavirus*	5 × 10^6^ (5 × 10^5^–5 × 10^6^)	4 (2–4)	>256 (>256–>256)	256 (256–>256)	>256 (>256–>256)	32 (32–32)
AUHB219	NDM	ST773	vB_PaerM_AttikonH10	*Phikzvirus*	5 × 10^7^ (5 × 10^7^–5 × 10^8^)	2 (1–2)	>256 (>256–>256)	128 (128–256)	>256 (>256–>256)	16 (8–16)
AUHB220	NDM	ST773	vB_PaerM_AttikonH7	*Pbunavirus*	5 × 10^6^ (5 × 10^4^–5 × 10^6^)	8 (4–8)	8 (8–8)	256 (256–>256)	8 (8–8)	8 (4–8)
AUHB222	NDM	ST308	vB_PaerM_AttikonH7	*Pbunavirus*	5 × 10^6^ (5 × 10^5^–5 × 10^7^)	2 (2–2)	>256 (>256–>256)	128 (128–256)	>256 (>256–>256)	32 (32–32)
AUHB174	VIM	ST235	vB_PaerM_AttikonH2	*Pakpunavirus*	5 × 10^6^ (5 × 10^6^–5 × 10^7^)	4 (2–4)	16 (16–16)	32 (16–32)	32 (16–32)	32 (32–32)
AUHB175	VIM	ST235	vB_PaerM_AttikonH2	*Pakpunavirus*	5 × 10^7^ (5 × 10^6^–5 × 10^7^)	4 (2–4)	8 (8–16)	16 (16–32)	32 (16–32)	32 (32–64)
AUHB183	VIM	ST395	vB_PaerM_AttikonH7	*Pbunavirus*	5 × 10^7^ (5 × 10^6^–5 × 10^8^)	4 (2–4)	64 (64–64)	16 (16–32)	64 (64–128)	16 (8–16)
AUHB186	VIM	ST235	vB_PaerM_AttikonH2	*Pakpunavirus*	5 × 10^9^ (5 × 10^9^–5 × 10^9^)	4 (4–4)	32 (16–32)	128 (64–128)	128 (128–128)	32 (32–32)
AUHB187	VIM	ST235	vB_PaerP_AttikonH4	*Phikmvvirus*	5 × 10^8^ (5 × 10^7^–5 × 10^8^)	4 (2–4)	128 (128–256)	256 (256–256)	64 (64–64)	16 (16–16)

^a^ NDM: new Delhi metallo-β-lactamase, VIM: Verona Integron borne metallo-β-lactamase.

**Table 2:** Median (range among strains ad replicates) MIC of antibiotics (MIC_A_) and phages (MIC_P_) of 5 antibiotics and 5 phages alone and in synergistic, additive, and antagonistic combinations, as well as the FICis against 10 MBL-producing *Pseudomonas aeruginosa* isolates using the checkerboard assay.

Antibiotic (No. of Strains)	Interactions	FICi ^a^	Monotherapy		Combination ^b^	
	(No. of Strains) ^phage^		MIC_A_ (mg/L)	MIC_P_ (PFU/mL)	MIC_A_ (mg/L)	MIC_P_ (PFU/mL)
Colistin (10)	Synergistic (4) ^1,3^	0.23 (0.13–0.23) **	4 (2–8)	5 × 10^7^ (5 × 10^5^–5 × 10^7^)	0.25 (0.125–2) **	5 × 10^6^ (5 × 10^1^–5 × 10^6^) *
	Additive (4) ^2,3,4,5^	1.5 (1.5–4)	4 (1–4)	5 × 10^7^ (5 × 10^5^–5 × 10^9^)	2 (0.5–8)	5 × 10^7^ (5 × 10^2^–5 × 10^9^)
	Antagonistic (2) ^1^	10.5 (10–11)	4 (1–4)	5 × 10^7^ (5 × 10^6^–5 × 10^8^)	2 (0.5–4)	5 × 10^8^ (5 × 10^3^–5 × 10^8^)
Ciprofloxacin (10)	Synergistic (5) ^1,2,3,4^	0.25 (0.04–0.35) *	32 (16–256)	5 × 10^7^ (5 × 10^6^–>5 × 10^9^)	4 (4–64) *	5 × 10^6^ (5 × 10^1^–5 × 10^9^) ***
	Additive (1) ^5^	1.5 (1.5–2)	256 (128–256)	5 × 10^5^ (5 × 10^5^–5 × 10^6^)	128 (128–128)	5 × 10^5^ (5 × 10^5^–5 × 10^6^)
	Antagonistic (4) ^1,3^	1000 (11–1001) *	256 (128–256)	5 × 10^6^ (5 × 10^4^–5 × 10^9^)	256 (128–256)	5 × 10^9^ (5 × 10^6^–>5 × 10^9^) *
Meropenem (10)	Synergistic (4) ^1,2,5^	0.12 (0.10–0.35) **	>256 (64–>256)	5 × 10^6^ (5 × 10^5^–5 × 10^8^)	8 (4–16) **	5 × 10^5^ (5 × 10^3^–5 × 10^7^) ***
	Additive (4) ^1,3,4^	1.25 (1–2)	16 (8–256)	5 × 10^8^ (5 × 10^5^–5 × 10^9^)	8 (8–128)	5 × 10^8^ (5 × 10^1^–5 × 10^9^)
	Antagonistic (2) ^1^	10.5 (10–100)	128 (8–>256)	5 × 10^6^ (5 × 10^6^–5 × 10^7^)	32 (4–256)	5 × 10^8^ (5 × 10^7^–5 × 10^8^)
Aztreonam (10)	Synergistic (0)	NA	NA	NA	NA	NA
	Additive (7) ^1,2,3,4,5^	1.25 (1–4)	16 (4–32)	5 × 10^7^ (5 × 10^4^–5 × 10^9^)	16 (4–32)	5 × 10^5^ (5 × 10^0^–5 × 10^9^) *
	Antagonistic (3) ^1,3^	100.5 (10.5–1000)	32 (16–64)	5 × 10^6^ (5 × 10^5^–5 × 10^6^)	16 (8–16)	5 × 10^8^ (5 × 10^7^–>5 × 10^9^) *
Amikacin (10)	Synergistic (6) ^1,2,3,5^	0.23 (0.11–0.35) **	128 (16–>256)	5 × 10^8^ (5 × 10^5^–5 × 10^9^)	8 (4–64) **	5 × 10^7^ (5 × 10^3^–5 × 10^8^) ****
	Additive (2) ^1,4^	2 (2–2.1)	32 (8–64)	5 × 10^6^ (5 × 10^5^–5 × 10^8^)	64 (16–128)	5 × 10^2^ (5 × 10^1^–5 × 10^4^)
	Antagonistic (2) ^1,5^	55 (10.06–100)	>256 (>256–>256)	5 × 10^5^ (5 × 10^5^–5 × 10^5^)	32 (4–32)	5 × 10^7^ (5 × 10^6^–5 × 10^7^) *

NA: not applicable, FICi: fractional inhibitory concentration, ^1^: vB_PaerM_AttikonH7, ^2^: vB_PaerM_AttikonH10, ^3^: vB_PaerM_AttikonH2, ^4^: vB_PaerP_AttikonH4, ^5^: vB_PaerP_AttikonH5, *: *p* < 0.05, **: *p* < 0.01, ***: *p* < 0.001, ****: *p* < 0.0001. **^a^** FICis of synergistic and antagonistic combinations were compared with 0.5 and 4, respectively. **^b^** The MIC_A_ and MIC_P_ in combination were compared with the MIC_A_ and MIC_P_ alone.

**Table 3:** Percent of synergy, additivity, and antagonism of five antibacterial drugs in combination with five different phages against 10 MBL-producing *Pseudomonas aeruginosa* isolates using checkerboard assay.

Antibiotic (No. of Strains)	Synergy (%)	Additivity (%)	Antagonism (%)
Colistin (10)	40	40	20
Ciprofloxacin (10)	50	10	40
Meropenem (10)	40	40	20
Aztreonam (10)	0	70	30
Amikacin (10)	60	20	20

## Data Availability

The original contributions presented in this study are included in the article. Further inquiries can be directed to the corresponding author.
